# Expression of Concern: Capsaicin inhibits migration and invasion via the AMPK/NF-kB signaling pathway in esophagus sequamous cell carcinoma by decreasing matrix metalloproteinase-9 expression

**DOI:** 10.1042/BSR-20190819_EOC

**Published:** 2020-07-02

**Keywords:** AMPK, Capsaicin, cell invasion, cell migration, ESCC

The Editorial Office has been made aware of potential issues surrounding the scientific validity of this paper, hence has issued an expression of concern to notify readers whilst the Editorial Office investigates.

